# Moderate to severe liver siderosis and raised AST are independent risk factors for vitamin D insufficiency in β-thalassemia patients

**DOI:** 10.1038/s41598-020-78230-4

**Published:** 2020-12-03

**Authors:** Hadi Darvishi-Khezri, Hossein Karami, Mohammad Naderisorki, Mohammad Zahedi, Alireza Razavi, Mehrnoush Kosaryan, Aily Aliasgharian

**Affiliations:** 1 Thalassemia Research Center (TRC), Hemoglobinopathy Institute, Mazandaran University of Medical Sciences, Sari, Mazandaran Iran; 2 Department of Laboratory Sciences, School of Allied Medical Science, Student Research Committee, Mazandaran University of Medical Sciences, Sari, Iran; 3 Student Research Committee, Mazandaran University of Medical Sciences, Sari, Iran

**Keywords:** Endocrinology, Medical research, Risk factors, Endocrine system and metabolic diseases, Haematological diseases

## Abstract

Numerous problematic disorders such as vitamin D (Vit-D) deficiency subsequent to large iron loading can be developed in patients with β-thalassemia. The study aimed to estimate Vit-D insufficiency and its risk factors in patients with β-thalassemia. In this multicenter and observational study, all β-thalassemia patients, who referred to 14 hospital-based thalassemia divisions or clinics in Mazandaran province, Iran were included in the study. The data belong to December 2015 until December 2019. The study population was made of transfusion dependent thalassemia (TDT) and non-transfusion-dependent thalassemia (NTDT) patients. Serum levels of 25-OHD3 have been measured by high performance liquid chromatography (HPLC) method as ng/mL. Demographic and clinical information along with some biological tests, as well as the results of T2*-weighted magnetic resonance imaging were analyzed. Of 1959 registered patients, 487 (24.9%) patients had Vit-D-related data. The prevalence of Vit-D insufficiency (< 30 ng/mL) was 41.9, 95% CI 37.5–46.3. The adjusted risks of moderate to severe liver siderosis and raised AST (aspartate aminotransferase) for Vit-D insufficiency (< 30 ng/mL) were 2.31, 95% CI 1.38–3.89 and 2.62, 95% CI 1.43–4.79, respectively. The receiver operating characteristic (ROC) curve analysis showed that the predictive accuracy of ferritin for Vit-D insufficiency status was 0.61, 95% CI 0.54–0.68 with a cutoff point of 1,078 ng/mL (*P* = 0.03, sensitivity 67%, specificity 49%, positive predictive value [PPV] 47% and negative predictive value [NPV] 68%). In spite of the national programs for treating Vit-D deficiency and our previous efforts for giving supplements to all patients, Vit-D insufficiency/deficiency is still common in our patients. Also, moderate to severe liver siderosis and raised AST were the independent risk factors for the Vit-D insufficiency.

## Introduction

β-Thalassemia, a congenital defect in the synthesis of the β-globin chains of the hemoglobin, is the most common genetic disease worldwide. It is estimated that 3% of the world population carries one of β-thalassemia phenotype characteristics^1,2^. Based on data obtained from the Thalassemia Association in Iran, the number of patients with thalassemia in 2005 was 18,616, and this number was enhanced to 20,000 in 2016. The majority of thalassemia patients have been scattered in the Mazandaran and Fars provinces^3–5^.

Low hemoglobin levels in patients with β-thalassemia major—so-called transfusion-dependent β-Thalassemia (TDT)—cause a need to chronic blood transfusion in the rest of their life^6^. Every blood unit includes near 200–250 mg iron^7–9^. Although the repeated blood transfusion plays a life-saving role in thalassemic patients, an iron toxicity would happen in these cases^10–12^. Many problematic disorders such as vitamin D (Vit-D) deficiency subsequent to large iron loading can be developed in TDT patients. The deficiency in Vit-D is associated with rickets/ osteomalacia and also a decrease in heart activity, muscle fatigue and insulin resistance^5,13,14^. Moreover, Vit-D deficiency is correlated with numerous mental and physical complications^15–21^.

Checking serum levels of 25-OHD3 is not recommended for general population due to the cost. In thalassemia wards, however, serum 25-OHD3 would be measured at least yearly and Vit-D supplements are being administered according to the results. To the best of our knowledge, there has not been any study in Iran aiming to investigate Vit-D status in patients with β-thalassemia with such a sample size. Based on the role of genetic diversities in the incidence of thalassemic complications^22^, and because of the lack of enough studies in Iranian population, the current study aimed to estimate Vit-D insufficiency and its risk factors in patients with β-thalassemia.

## Materials and methods

### Ethics statement

Mazandaran Thalassemia Registry (MTR) was approved by the Vice Chancellor of Research and Technology, the Ethics Committee and Institutional Review Board (IRB) of Mazandaran University of Medical Sciences in 2015. The university vouched the study protocol and all methods were performed in accordance with the relevant guidelines and regulations. Before data registration, all patients got informed and oriented by all physicians and care providers who were working with these patients throughout the province. The informed written consent was obtained from all the participants in the current study. All patients were assured that their information will be confidentially kept. The online website is available at thr.mazums.ac.ir.

### Study design

The thalassemia registry is an online data bank for a quick and on time assessment of epidemiological and clinical aspects of patients with β-thalassemia in the province, working since 2015. This multicenter and observational study was based on recorded data and a census method. All patients with β-thalassemia, who referred to 14 hospital-based thalassemia divisions or clinics under Mazandaran University of Medical Sciences, between December 2015 and December 2019 were included into the study. The thalassemia research center (TRC) of Bu-Ali Sina hospital was the core center to establish the study.

### Patients and data collection

The study population was made of TDT and non-transfusion-dependent thalassemia (NTDT) patients. All patients were diagnosed and documented by their responsible physicians based on the results of CBC (complete blood count) after birth, hemoglobin electrophoresis and genetic test for mutations of the β-globin gene. Transfusion dependent thalassemia cases were on a regular red-cell transfusion.

The information sources comprise demographic, bio-clinical and therapeutic data in thalassemic wards in the north of Iran (n = 1959). The patients' information was extracted from the MTR with ethical points using a self-made checklist. The checklist contains variables, including demographic and clinical features, transfusion dependency and information about iron chelation therapy. Also, we used medical history and laboratory data such as AST (aspartate aminotransferase), ALT (alanine aminotransferase), serum ferritin, calcium, phosphor, parathyroid hormone (PTH) and use of Vit-D supplementation.

#### Magnetic resonance imaging (MRI)

A MRI without contrast was performed on a 1.5 T MR scanner (Symphony; Siemens, Germany) to calculate the T2*-weighted magnetic resonance imaging (MRI T2*, millisecond [ms]) for all patients. Cardiac MRI was carried out based on the Brompton protocol^23^. To measure myocardial T2*-weighted, scans were coordinated to the cardiac cycle by use of standard electrocardiography gating. A standard radio frequency body coil was also applied in all measurements. The region of interest (ROI) was determined in mid-ventricle. The center of the liver was imaged at 12 diverse echo times (1.29 to 23 ms, increasing in 2.2 ms increments) by a single trans-axial slice^24^.

The results of heart and liver MRI T2* were registered as normal to severe^25^. Cardiac siderosis was characterized by cardiac MRI T2* index, a myocardial loading, as follows: ≥ 20 ms (normal), 19.99–14 ms (mild), 13.99–10 ms (moderate) and < 10 ms (severe). The classification of hepatic siderosis severity was based on hepatic MRI T2* index, a hepatic loading, as normal (≥ 6.3 ms), mild (6.3–2.80 ms), moderate (2.79–1.4 ms) and severe (< 1.4 ms)^25^.

#### Echocardiography

According to the American Society of Echocardiography, a transthoracic Echocardiography, M-mode, 2D, color Doppler was used to prepare the echocardiograms and GE Vivid S5 echocardiographic machine (General Electric, Boston, MA, USA) at rest and in supine and left lateral decubitus position. To measure the left ventricular ejection fraction (LVEF), the biplane Simpson technique was applied^26,27^. Pulmonary artery pressure (PAP) was also valued by use of the Bernoulli equation to estimate the pressure gradient between the right ventricle and the right atrium^28^. Reduced LVEF was also considered as LVEF lower than 55%^29^.

### Biochemical parameters

Serum levels of 25-OHD3 have been measured by high performance liquid chromatography (HPLC) method as ng/mL. A categorical scale was also used for Vit-D insufficiency as follows: insufficient/ deficient (< 30 ng/mL), insufficient (30–20 ng/mL), deficient (< 20 ng/mL), moderately deficient (20–10 ng/mL) and severe deficiency (< 10 ng/mL)^30,31^. The serum AST and ALT levels higher than 40 and 56 U/L were defined as abnormal ranges^32^. Serum ferritin 800 ≥ ng/mL and 2000 ≥ ng/mL were demarcated as iron overload condition in NTDT and TDT cases, respectively^33,34^.

### Statistical analysis

All statistical procedures were performed by SPSS software package (version 20.0, SPSS Inc., Chicago, IL, USA) and STATA version 13 (StataCorp, College Station, TX, USA). Data have been expressed as mean ± standard deviation (M ± SD) or number (percent). The normal distribution of quantitative variables was checked through histogram plot and Shapiro–Wilk test. We utilized the independent student's t- and chi-square tests to compare the variables between two groups, insufficient versus non-insufficient in 25-OHD3 level. The risk of different grades of Vit-D insufficiency was computed as crude and adjusted. To calculate the adjusted odds ratio (OR), the effects of multiple potential confounders including age, gender, transfusion dependency, calcitriol, Vit-D and calcium supplementations were controlled. The correlation between 25-OHD3 and serum ferritin was estimated using Pearson correlation coefficient. A logistic regression model was lastly applied to sort the risk factors based on their strength in this study. The goodness of fit of the model was assessed by means of Hosmer and Lemeshow test. We also checked multicollinearity between independent variables for the final model with the LMCOL command, based on VIF index (variance inflation factor). A receiver operating characteristic (ROC) curve analysis was operated to determine an optimal statistically significant threshold value of ferritin in discriminating Vit-D insufficiency. The prevalence of Vit-D insufficiency grades, with 95% confidence interval (CI), were also estimated using the binomial exact method by STATA 13 software. A *P* value lower than 0.05 was chosen as the threshold of statistical significance. A correction on the estimated ORs was performed in our study by an online web calculator (https://clincalc.com/Stats/ConvertOR.aspx).

## Results

Out of 1959 registered data, 487 patients had Vit-D-related data in the thalassemia registry (24.9%). Basic characteristics, clinico-biological and therapeutic status of β-thalassemia patients are also shown in Table 1.

**Table 1:** Basic characteristics, clinico-biological and therapeutic status of β-thalassemia patients (n = 487).

Variable	25-OHD3 < 30 ng/mL		*P* value
	yes (n = 204)	no (n = 283)	
Age, year	32.7 ± 8.25	33.1 ± 9.16	0.7^ƒ^
**Gender**			
Male	98 (48.04)	107 (37.6)	0.3^ǃ^
Female	106 (51.96)	176 (62.19)	
Weight, kg	57.4 ± 8.83	58.2 ± 10.5	0.4^ƒ^
Transfusion dependency	177 (86.8)	236 (83.4)	0.4^ǃ^
Hb, gr/dl	8.84 ± 1.10	9.27 ± 5.06	0.2^ƒ^
Raised ALT	25 (12.2)	24 (8.5)	0.10^ǃ^
Raised AST	44 (21.6)	26 (9.2)	**< 0.001**^ǃ^
Ferritin, ng/mL	2217 ± 2172	1593 ± 1653	**0.001**^†^
Deferoxamine	111 (54.4)	152 (53.7)	0.9^ǃ^
Deferoxamine, injection number per week	4.97 ± 1.28	4.99 ± 1.13	0.9^ƒ^
Deferasirox	57 (27.9)	76 (26.8)	0.9^ǃ^
Deferasirox, mg/day	1250 ± 546	1229 ± 486	0.8^ƒ^
Deferiprone	74 (36.3)	89 (31.4)	0.3^ǃ^
Deferiprone, pill number (500 mg) /day	5.23 ± 1.58	5.14 ± 1.47	0.7^ƒ^
Deferoxamine plus deferiprone	69 (33.8)	80 (28.3)	0.2^ǃ^
Deferasirox plus deferiprone	6 (2.9)	5 (1.8)	0.4^ǃ^
Calcium, 500 mg/day	151 (74.0)	216 (76.3)	**0.04**^ǃ^
Calcitriol	77 (37.7)	122 (43.1)	0.1^ǃ^
Vit-D supplementation	141 (69.1)	189 (66.8)	0.8^ǃ^
PTH	0.96 ± 0.19	0.94 ± 0.23	0.4^ƒ^

Data are presented as mean ± standard deviation or number (%).

*ALT* aspartate aminotransferase, *AST* alanine aminotransferase, *PTH* parathormone hormone.

^ǃ^*P* value was obtained by Chi-square test.

^ƒ^*P* value was calculated by independent samples t-test.

^†^*P* value was calculated by *Mann*–*Whitney U* test.

The mean ± SD of Vit-D was 38.31 ± 41.20 ng/mL (3 to 786, median = 32.20 ng/mL). The prevalence of Vit-D insufficiency (< 30 ng/mL) in all β-thalassemia patients was 41.9, 95% CI 37.5–46.3. The difference in the prevalence of Vit-D insufficiency between TDT and NTDT patients was significantly found in the severe Vit-D deficiency category. The results of a subgroup analysis for the prevalence of Vit-D insufficiency in all grades according to blood transfusion dependency are shown in Table 2.

**Table 2:** Status of Vit-D in β-thalassemia patients according to blood transfusion dependency.

25-OHD3 level, ng/mL	Status	N	Prevalence with 95% CI			*P* value
			All	TDT	NTDT	
< 30	Insufficient/deficient	204	41.9, 37.5–46.3	42.7, 38.1–47.6	36.6, 25.1–48.1	0.4
30–20	Insufficient	108	27.6, 23.2–32.1	28.1, 23.2–32.9	25.1, 13.7–36.3	0.7
< 20	Deficient	96	19.7, 16.2–23.3	20.6, 16.7–24.6	15.5, 06.9–24.1	0.4
20–10	Moderately deficient	69	15.1, 11.7–18.3	15.1, 11.4–18.6	15.5, 6.87–24.1	0.8
< 10	Severe deficiency	27	5.54, 3.50–7.58	6.54, 4.14–8.93	-	**0.02**

*25-OHD3* 1,25-dihydroxyvitamin D3, *n* number, *CI* confidence interval, *TDT* Transfusion dependent thalassemia, *NTDT* non-transfusion-dependent thalassemia.

*P* values were estimated by Chi-square test after the comparison of Vit-D insufficiency grades between TDT and NTDT patients.

Of 19 registered data regarding LVEF, the percentage of reduced LVEF in Vit-D insufficient patients (< 30 ng/mL) was 60% (three cases, n = 5) compared with 28.6% (four cases, n = 14) among non-Vit-D insufficient patients (OR 3.75, 95% CI 0.44–31.62, *P* = 0.21). The mean ± SD of LVEF in Vit-D insufficient patients (< 30 ng/mL) versus non- insufficient individuals was 51.0 ± 8.94% and 53.9 ± 5.25%, respectively (*P* = 0.4). The difference of PAP among patients with Vit-D insufficiency (< 30 ng/mL) and without Vit-D insufficiency was not significant (M ± SD, 38.3 ± 2.89 mmHg versus 34.2 ± 9.67 mmHg; *P* = 0.5, respectively.

The OR of iron overload (ferritin 800 ≥ ng/mL) for Vit-D insufficiency (< 30 ng/mL) in cases with NTDT was 1.84, 95% CI 1.17–2.90. In TDT patients, the risk of iron overload (ferritin 2000 ≥ ng/mL) was calculated 1.5, 95% CI 0.52–4.34 for Vit-D insufficiency.

The adjusted risks of moderate to severe liver siderosis and raised AST for Vit-D insufficiency (< 30 ng/mL) were 2.31, 95% CI 1.38–3.89 and 2.62, 95% CI 1.43–4.79, respectively. The adjusted risk in patients with raised AST for severe Vit-D insufficiency (< 10 ng/mL) has been significantly valued at 6.49, 95% CI 2.39–17.62. The potential risk factors for Vit-D insufficiency in β-thalassemia patients are shown in Table 3.

**Table 3:** Potential risk factors for Vit-D insufficiency in β-thalassemia patients (n = 487).

Variable	OR with 95% CI									
	< 30 ng/mL		30–20 ng/mL		< 20 ng/mL		20–10 ng/mL		< 10 ng/mL	
	Model ^1^	Model ^2^	Model ^1^	Model ^2^	Model ^1^	Model ^2^	Model ^1^	Model ^2^	Model ^1^	Model ^2^
Cardiac siderosis	1.50, 0.92–2.44	1.56, 0.90–2.69	1.39, 0.76–2.53	1.62, 0.81–3.23	1.50, 0.83–2.68	1.30, 0.68–2.50	1.46, 0.75–2.84	1.28, 0.61–2.69	1.51, 0.55–4.15	1.12, 0.35–3.60
Moderate to severe cardiac siderosis	1.51, 0.77–2.94	1.33, 0.64–2.74	1.16, 0.49–2.77	1.18, 0.45–3.06	1.84, 0.86–3.92	1.35, 0.59–3.08	1.84, 0.78–4.32	1.51, 0.61–3.75	1.65, 0.46–5.99	0.93, 0.19–4.71
Liver siderosis	1.34, 0.84–2.14	1.33, 0.76–2.32	1.05, 0.60–1.84	0.97, 0.49–1.93	1.81, 0.95–3.44	2.02, 0.95–4.30	1.84, 0.88–3.86	1.79, 0.76–4.23	1.61, 0.52–5.01	2.36, 0.60–9.26
Moderate to severe liver siderosis	2.27, **1.42**–**3.58**	2.31, **1.38**–**3.89**	1.92, **1.09**–**3.38**	2.06, **1.07**–**3.95**	2.24, **1.29**–**3.90**	2.31, **1.23**–**4.33**	2.30, **1.23**–**4.32**	2.46, **1.22**–**4.97**	1.84, 0.70–4.79	1.41, 0.43–4.58
Iron overload^a^	1.90, **1.24**–**2.91**	1.75, **1.08**–**2.82**	1.64, 0.97–2.78	1.35, 0.74–2.49	1.93, **1.16**–**3.22**	2.22, **1.24**–**3.97**	1.72, 0.95–3.10	2.24, **1.14**–**4.37**	2.37, 0.99–5.62	1.98, 0.77–5.14
Raised ALT	1.64, 0.90–2.99	1.68, 0.85–3.30	1.22, 0.55–2.66	1.13, 0.44–2.93	2.03, **1.04**–**3.95**	2.30, **1.10**–**4.81**	1.22, 0.51–2.90	1.40, 0.55–3.55	4.72, **1.87**–**11.94**	4.91, **1.74**–**13.81**
Raised AST	3.08, **1.81**–**5.26**	2.62, **1.43**–**4.79**	2.42, **1.26**–**4.64**	1.68, 0.75–3.76	2.93, **1.66**–**5.19**	3.35, **1.77**–**6.34**	2.07, **1.05**–**4.10**	2.30, **1.08**–**4.90**	5.46, **2.26**–**13.16**	6.49, **2.39**–**17.62**

*ALT* aspartate aminotransferase, *AST* alanine aminotransferase, *OR* odds ratio, *CI* confidence interval.

^a^Serum ferritin 2000 ≥ ng/mL was considered as iron overload for the study population, including both transfusion dependent and non-transfusion-dependent thalassemia patients.

Model ^1^: unadjusted odds ratios .

Model ^2^: ORs were adjusted according to age, gender, transfusion dependency, calcitriol, Vit-D and calcium supplementations.

After converting ORs to risk ratio (RR) index, the modified risk of Vit-D insufficiency (< 30 ng/mL) for patients with cardiac siderosis changed to a risk of 18% (RR = 1.18). The adjusted risk of 16% was also found for Vit-D insufficiency (< 30 ng/mL) in cases who had moderate to severe cardiac siderosis, RR = 1.16. The risk of Vit-D insufficiency (< 30 ng/mL), 12% raised in patients with liver siderosis after a bias correction (RR = 1.12). A 31% escalation in the risk of Vit-D insufficiency (< 30 ng/mL) was computed for patients with moderate to severe liver siderosis (RR = 1.31). In iron overloaded cases (ferritin 2000 ≥ ng/mL), the risk of Vit-D insufficiency (< 30 ng/mL) decreased to 25% after the correction of upward bias (RR = 1.25). The modified risk of Vit-D insufficiency (< 30 ng/mL) for patients with raised ALT and cases with raised AST changed to the risk of 19% and 39%, respectively (RR = 1.19 and RR = 1.39).

The multivariate logistic regression analysis presented a significant association between raised AST and also moderate to severe liver siderosis with Vit-D insufficiency (< 30 ng/mL). The results of the multivariate logistic regression model have been provided in Table 4. The ROC curve analysis showed that the predictive accuracy of ferritin for Vit-D insufficiency status was 0.61, 95% CI 0.54–0.68 with a cutoff point of 1,078 ng/mL (*P* = 0.03, sensitivity 67%, specificity 49%, positive predictive value [PPV] 47% and negative predictive value [NPV] 68%). There was a significant negative association between 25-OHD3 and serum ferritin (r =  − 0.2; *P* < 0.01).

**Table 4:** The results of multivariate logistic regression analysis for the Vit-D insufficiency (< 30 ng/mL) in β-thalassemia patients (n = 487).

Variable	B	SE	Wald	Adjusted OR, 95% CI	*P* value
Raised AST	1.25	1.68	6.70	3.48, **1.35**–**8.97**	**0.01**
Moderate to severe liver siderosis	0.83	0.67	8.30	2.30, **1.30**–**4.06**	**0.004**
Iron overload^a^	0.12	0.33	0.15	1.124, 0.62–2.02	0.69
Raised ALT	− 0.79	0.25	1.98	0.45, 0.15–1.36	0.16
Constant	− 1.76	0.06	21.24	0.17, 0.08–0.36	**< 0.001**

*ALT* aspartate aminotransferase, *AST* alanine aminotransferase, *OR* odds ratio, *CI* confidence interval, *SE* standard error.

^a^Serum ferritin 2000 ≥ ng/mL was considered as iron overload for the study population, including both transfusion dependent and non-transfusion-dependent thalassemia patients.

The logistic regression model was statistically significant, *X* ^2^ (4, n = 487) = 22.2, *P* < 0.001. Log-likelihood was 362.7, and the model explained between 7.4% (Cox & Snell *R*^*2*^) and 10.0% (Nagelkerke *R*^*2*^) of the variance in Vit-D insufficiency and also correctly classified 62.5% of cases. Goodness of fit for the model was appropriate (Hosmer and Lemeshow χ^2^ = 1.99, *P* = 0.73).

## Discussion

This study was designed to evaluate the prevalence and risk factors of Vit-D insufficiency in β-thalassemia patients. Although the majority of patients—roughly 68 percent—received Vit-D supplementation, the prevalence of Vit-D insufficiency is not ignorable. The prevalence of the majority of Vit-D insufficiency was highly detected among TDT patients. Cardiac siderosis, moderate to severe cardiac siderosis, liver siderosis, moderate to severe liver siderosis, iron overload (ferritin 2000 ≥ ng/mL) and the raise of ALT and AST levels were introduced as the risk factors for Vit-D insufficiency in this population. After controlling potential confounders, the multivariate logistic regression analysis presented that raised AST and moderate to severe liver siderosis as the outstanding risk factors had the maximum strength of association with Vit-D insufficiency in β-thalassemia patients.

Some evidence has shown that Vit D deficiency has been seen in 12.8 to 82% of TDT patients^35,36^. Progressive accumulation of iron^37^, parenchymal hemosiderosis^38^, insufficient hydroxylation in the liver^39^, malabsorption of Vit-D intestinal, and impaired synthesis of Vit D in the skin subsequent to jaundice^37^ have been reported as the plausible causes of Vit-D insufficiency in TDT patients. Elevated serum iron levels ensues from blood transfusions and hyperabsorption of dietary iron by the gastrointestinal tract—especially in NTDT alongside reduced hepcidin as the hepatic peptide hormone regulating iron homeostasis^40^—are conducive to the iron siderosis in several organs such as tissue, cardiac, liver and also endocrine system glands, which harm their natural physiology. Subsequently, endocrinopathy and Vit-D deficiency as the adverse consequences resulting from superfluous iron burden can occur in patients with TDT^41,42^.

Previous studies have shown that Vit-D levels were significantly reduced in thalassemia patients who had chronic blood transfusions^31,37,43^. In current study, the greatest prevalence of Vit-D insufficiency belongs to TDT, except one subgroup of Vit-D insufficiency (20 to 10 ng/mL). In other words, the prevalence of the majority of Vit-D insufficiency was highly detected among TDT patients.

Repeated blood transfusions can cause secondary iron overload conditions which may disturb the 25-hepatic hydroxylation and synthesis of Vit D, causing Vit D deficiency^31^. Also, an iron deposition in the intestinal epithelium may interrupt the gastrointestinal absorption of Vit-D which can contribute to develop a comparatively resistant to Vit D supplementations in β-thalassemia patients^44^. It has been stated that the darkening of the skin due to iron siderosis seems to interfere with Vit-D synthesized in the skin in TDT patients^44^. Given this, we speculate that iron overload also contributed to Vit-D deficiency in these patients.

Recently, the results of a cross-sectional on 61 patients with TDT have shown that there has been an relationship between Vit-D deficiency and cardiac MRI T2* in these patients. The mean (range) of 25-OHD3 levels in patients with cardiac siderosis was 15.9 ng/mL (11.7 to 20.0 ng/mL) compared to 20.2 ng/mL (15.85 to 22.3 ng/mL) in patients without siderosis (*P* = 0.06). The reported prevalence of Vit-D deficiency was also 50.8%^45^. In another cross-sectional study on 40 TDT patients, there was no correlation between serum 25-OHD3 and liver and cardiac MRI T2*. The mean of 25-OHD3 serum levels was 16.1 ± 8.1 ng/dl^5^.

Patients with moderate to severe liver siderosis and/or iron-overload had a greater tendency to Vit-D insufficiency than those without moderate to severe liver siderosis and/or iron-overload. Transfusional iron burden in TDT patients leads to an increase in ferritin and the formation of iron siderosis in liver^46^. In other findings confirmed in β-thalassemia population, serum ferritin levels are significantly higher in patients with Vit-D deficiency^35,37,44^. Excessive accumulation of iron can help to promote Vit-D deficiency resultant a disturbance in Vit-D-PTH axis.

A study aimed to examine the association of hepatic siderosis with Vit-D-PTH axis in TDT patients. That results demonstrated that ferritin levels of > 2500 μg/L were an independent risk factor for Vit-D insufficiency in the patients with TDT (OR 5.3, 95% CI 2.3–12.3; *P* < 0.01)^46^. In a recent study, the patients deprived of Vit-D supplements with ferritin levels of > 9000 ng/mL had multiple endocrinopathies including osteoporosis and low levels of 25-OHD3 (17.1 ± 7.9 ng/mL)^47^.

In a case–control study^46^, in a case group of 158 TDT patients with an average of 26.5 ± 1.1 nmol /L for 25-OHD3, Vit D deficiency had a 8.8-fold increase in the patients with liver iron concentration (LIC) above 7 mg/g dry weight (OR 8.8, 95% CI 3.5–10.3; *P* < 0.01). The prominent role of iron in the overgeneration of reactive oxygen specious (ROS) and the exacerbation of oxidative stress through Fenton and Haber–Weiss reactions are well-documented^48,49^. Indeed, iron burden can be noxious for vital organs and can expedite the endocrine disorders via an overproduction of ROS, disrupting the ferroportin-hepcidin axis. As liver is a target organ for the iron deposition, the inception of iron siderosis can begin in hepatic tissue in overloaded patients. Eventually, the progressive iron excess and a deleterious iron deposition in the liver can lead to structural and functional damage to hepatocytes through fibrosis^46^.

Our findings revealed that the patients with increased levels of ALT and AST had a greater risk of severe Vit-D insufficiency versus those without raise of ALT and/or AST levels. Liver damage due to iron overload in the liver can increase liver enzymes such as ALT and AST^50^. It has been shown that the reduced levels of 25-OHD3 can increase the likelihood of raising ALT and AST levels in β-thalassemia patients^51^. In a study, the elevated levels of ALT (> 50 IU/L) were significantly connected to Vit-D insufficiency in patients with β-thalassemia (OR 9.7, 95% CI 4.0–23.5; *P* < 0.001)^46^. Since the serum ALT levels might be dependent on LIC, a prolonged hepatic iron deposition may be the main cause of decreased 25-OHD3 level in blood circulation among patients with β-thalassemia^46^.

Regarding some surveys, Vit-D deficiency increases PTH and hyperparathyroidism^52^ and PTH affects the myocardium both directly and indirectly through G protein-coupled receptors, causing left ventricular hypertrophy and an increased risk of heart failure^53^. On the other hand, the transfer of iron to the heart myocytes and iron overload are performed by cardiac L-type voltage-dependent calcium channels (LVDCCs) which induce heart dysfunction^45^. It should also be noted that Vit-D deficiency causes the production of inflammatory cytokines, creating oxidative stress, and accelerating the process of fibrosis of the heart muscle cells. All of these mechanisms are associated with a reduction in LVEF in thalassemia patients with Vit-D deficiency^54^. In our findings, the risk of decreased LVEF in the cases with Vit-D insufficiency was near twofold higher than those who did not have Vit-D insufficiency. However, more well-defined and large studies to investigate the relationship between reduced LVEF and Vit-D insufficiency in β-thalassemia patients are required in the future.

### Study limitations

There have been several limitations in current study. These limitations include other factors that can effect on Vit-D status, comprising sun exposure and outdoor activity that have not been evaluated. In this study, the effects of other nutritional deficiencies on heart function were not investigated. The measure time of Vit-D test and doing cardiac and liver MRI and also echocardiography were not exactly concurrent. Moreover, the ferritin and liver enzyme levels may be affected by the possible existing infectious diseases at the test time in our population. Finally, the quantitative values of LIC, heart and liver MRI T2* were not considered to be registered in our data registry for further analyses.

## Conclusion

In spite of the national programs for treating Vit-D deficiency and our previous efforts for giving supplements to all patients, Vit-D insufficiency/deficiency is still common in our patients and needs more attention. Also, moderate to severe liver siderosis and raised AST were the independent risk factors for the Vit-D insufficiency in β-thalassemia.

## References

[1] Gregg X, Agarwal A, Prchal JT (2019). Concise Guide to Hematology.

[2] Origa R (2017). β-Thalassemia. Genet. Med..

[3] Habibzadeh F, Yadollahie M, Merat A, Haghshenas M (1998). Thalassemia in Iran; an overview. Arch. Iran. Med..

[4] Weatherall DJ (2018). The evolving spectrum of the epidemiology of thalassemia. Hematol. Oncol. Clin..

[5] Shaykhbaygloo R (2020). Correlation of cardiac and liver iron level with T2* MRI and vitamin D3 serum level in patients with thalassemia major. J. Blood Med..

[6] Lucarelli G, Gaziev J (2008). Advances in the allogeneic transplantation for thalassemia. Blood Rev..

[7] Cao A, Galanello R (2010). Beta-thalassemia. Genet. Med..

[8] Kontoghiorghes GJ, Pattichi K, Hadjigavriel M, Kolnagou A (2000). Transfusional iron overload and chelation therapy with deferoxamine and deferiprone (L1). Transfus. Sci..

[9] Taherahmadi H, Moradabadi AR, Arjomand Shabestari A, Nazari J, Kahbazi MK (2019). Antibiotic induced hemolytic anemia and thrombocytopenia among pediatric patients admitted to intensive care unit. Iran. J. Pediatr. Hematol. Oncol..

[10] Eghbali A, Taherahmadi H, Shahbazi M, Bagheri B, Ebrahimi L (2014). Association between serum ferritin level, cardiac and hepatic T2-star MRI in patients with major β-thalassemia. Iran. J. Pediatr. Hematol. Oncol..

[11] Chuansumrit A (2017). Effect of iron chelation therapy on glucose metabolism in non-transfusion-dependent thalassaemia. Acta Haematol..

[12] Kuo KH, Mrkobrada M (2014). A systematic review and meta-analysis of deferiprone monotherapy and in combination with deferoxamine for reduction of iron overload in chronically transfused patients with β-thalassemia. Hemoglobin.

[13] Jensen C (1998). High prevalence of low bone mass in thalassaemia major. Br. J. Haematol..

[14] Vogiatzi MG (2009). Bone disease in thalassemia: a frequent and still unresolved problem. J. Bone Miner. Res..

[15] Zahedi M, Razavi A, Sajjadi M, Nasirzadeh A (2019). The effect of vitamin D on depression in individuals. Int. J. Med. Rev..

[16] Yousif MM (2019). Associated vitamin D deficiency is a risk factor for the complication of HCV-related liver cirrhosis including hepatic encephalopathy and spontaneous bacterial peritonitis. Intern. Emerg. Med..

[17] Greenhagen RM, Frykberg RG, Wukich DK (2019). Serum vitamin D and diabetic foot complications. Diabet. Foot Ankle.

[18] Hattangdi-Haridas SR, Lanham-New SA, Wong WHS, Ho MHK, Darling AL (2019). Vitamin D deficiency and effects of vitamin D supplementation on disease severity in patients with Atopic Dermatitis: a systematic review and meta-analysis in adults and children. Nutrients.

[19] Hossain S (2019). Vitamin D and breast cancer: a systematic review and meta-analysis of observational studies. Clin. Nutr. ESPEN.

[20] Cuomo A (2019). Prevalence and correlates of vitamin D deficiency in a sample of 290 inpatients with mental illness. Front. Psychiatry.

[21] Ghaderi, A., Rasouli-Azad, M., Farhadi, M. H., Mirhosseini, N., Motmaen, M., Pishyareh, E. *et al.* Exploring the effects of vitamin D supplementation on cognitive functions and mental health status in subjects under methadone maintenance treatment. *J. Addict. Med.* **14**(1), 18–25 (2020). 10.1097/ADM.0000000000000550 31145174

[22] Singh K, Kumar R, Shukla A, Phadke SR, Agarwal S (2012). Status of 25-hydroxyvitamin D deficiency and effect of vitamin D receptor gene polymorphisms on bone mineral density in thalassemia patients of North India. Hematology.

[23] Anderson L (2001). Cardiovascular T2-star (T2*) magnetic resonance for the early diagnosis of myocardial iron overload. Eur. Heart J..

[24] Gholizadeh N (2012). Optimization of liver iron load assessment by pixel-based T2* MRI in thalassemic patients. Open J Radiol..

[25] Kosaryan, M., Rahimi, M., Zamanfar, D. & Darvishi-Khezri, H. Liver iron concentration is an independent risk factor for the prediabetic state in β-thalassemia patients. *Int. J. Diabetes Dev. Ctries.* **40**(2), 227–234 (2020).

[26] Schiller NB (1989). Recommendations for quantitation of the left ventricle by two-dimensional echocardiography. J. Am. Soc. Echocardiogr..

[27] Godara H (2013). The Washington Manual of Medical Therapeutics.

[28] Zipes DP, Libby P, Bonow RO, Mann DL, Tomaselli GF (2018). Braunwald’s Heart Disease E-Book: A Textbook of Cardiovascular Medicine.

[29] Meloni, A. *et al.* Biventricular reference values by body surface area, age, and gender in a large cohort of well-treated thalassemia major patients without heart damage using a multiparametric CMR approach. *J. Magn. Reson. Imaging.*10.1002/jmri.27169 (2020). 10.1002/jmri.27169 32311193

[30] Fahim FM, Saad K, Askar EA, Eldin EN, Thabet AF (2013). Growth parameters and vitamin D status in children with thalassemia major in upper Egypt. Int. J. Hematol. Oncol. Stem Cell Res..

[31] Fung EB, Aguilar C, Micaily I, Haines D, Lal A (2011). Treatment of vitamin D deficiency in transfusion-dependent thalassemia. Am. J. Hematol..

[32] Gowda S (2009). A review on laboratory liver function tests. Pan Afr. Med. J..

[33] Ekwattanakit S, Siritanaratkul N, Viprakasit V (2018). A prospective analysis for prevalence of complications in Thai nontransfusion-dependent Hb E/β-thalassemia and α-thalassemia (Hb H disease). Am. J. Hematol..

[34] Casale M (2015). Multiparametric cardiac magnetic resonance survey in children with thalassemia major: a multicenter study. Circ. Cardiovasc. Imaging.

[35] Vogiatzi MG (2009). Differences in the prevalence of growth, endocrine and vitamin D abnormalities among the various thalassaemia syndromes in North America. Br. J. Haematol..

[36] Soliman A, De Sanctis V, Yassin M (2013). Vitamin D status in thalassemia major: an update. Mediterr. J. Hematol. Infect. Dis..

[37] Napoli N (2006). Low serum levels of 25-hydroxy vitamin D in adults affected by thalassemia major or intermedia. Bone.

[38] Tzoulis P (2014). Prevalence of low bone mass and vitamin D deficiency in β-thalassemia major. Hemoglobin.

[39] Skordis N, Efstathiou E, Kyriakou A, Toumba M (2008). Hormonal dysregulation and bones in thalassaemia—an overview. PER.

[40] Nemeth E (2010). Hepcidin in β-thalassemia. Ann. N. Y. Acad. Sci..

[41] Goldberg EK, Neogi S, Lal A, Higa A, Fung E (2018). Nutritional deficiencies are common in patients with transfusion-dependent thalassemia and associated with iron overload. J. Food Nutr. Res. (Newark, Del.).

[42] Isik P (2014). Endocrinopathies in Turkish children with Beta thalassemia major: results from a single center study. Pediatr. Hematol. Oncol..

[43] Gaudio A (2019). Pathogenesis of thalassemia major-associated osteoporosis: a review with insights from clinical experience. J. Clin. Res. Pediatr. Endocrinol..

[44] Maleki, E., Daei, M. H. & Ebrahimi, P. The relationship between plasma level of 25-hydroxy vitamin D3 and plasma ferritin level in children with thalassemia major. *Afzalipour J. Clin. Res.* **1**(1), 9–18. 10.22122/ajcr.2016.44145 (2016).

[45] Dejkhamron P (2018). Vitamin D deficiency and its relationship with cardiac iron and function in patients with transfusion-dependent thalassemia at Chiang Mai University Hospital. Pediatr. Hematol. Oncol..

[46] Bajoria R, Rekhi E, Almusawy M, Chatterjee R (2019). Hepatic hemosiderosis contributes to abnormal vitamin D-PTH axis in thalassemia major. J. Pediatr. Hematol. Oncol..

[47] Kim MK (2013). Endocrinopathies in transfusion-associated iron overload. Clin. Endocrinol..

[48] Kehrer JP (2000). The Haber–Weiss reaction and mechanisms of toxicity. Toxicology.

[49] Nafady A (2017). Oxidative stress in pediatric patients with β thalassemia major. Egypt. J. Haematol..

[50] Salama KM (2015). Liver enzymes in children with beta-thalassemia major: correlation with iron overload and viral hepatitis. Open Access Maced. J. Med. Sci..

[51] Skaaby T (2014). Vitamin D status, liver enzymes, and incident liver disease and mortality: a general population study. Endocrine.

[52] Holick M, Binkley NC, Bischoff-Ferrari HA, Gordon CM, Hanley DA, Heaney RP (2011). evaluation, treatment, and prevention of vitamin D deficiency: an endocrine Society clinical practice guideline. J. Clin. Endocrinol. Metab..

[53] Brown SJ, Ruppe MD, Tabatabai LS (2017). The parathyroid gland and heart disease. Methodist DeBakey Cardiovasc. J..

[54] Rai V, Agrawal DK (2017). Role of vitamin D in cardiovascular diseases. Endocrinol. Metab. Clin..

